# Supplementary figures and images for: Utility of three-dimensional echocardiography for evaluating right ventricular size and function and ventricular myocardial deformation in repaired tetralogy of fallot
Source: PLoS One. 2026 Mar 17;21(3):e0344373. doi: 10.1371/journal.pone.0344373 (PMC12994780; doi:10.1371/journal.pone.0344373)

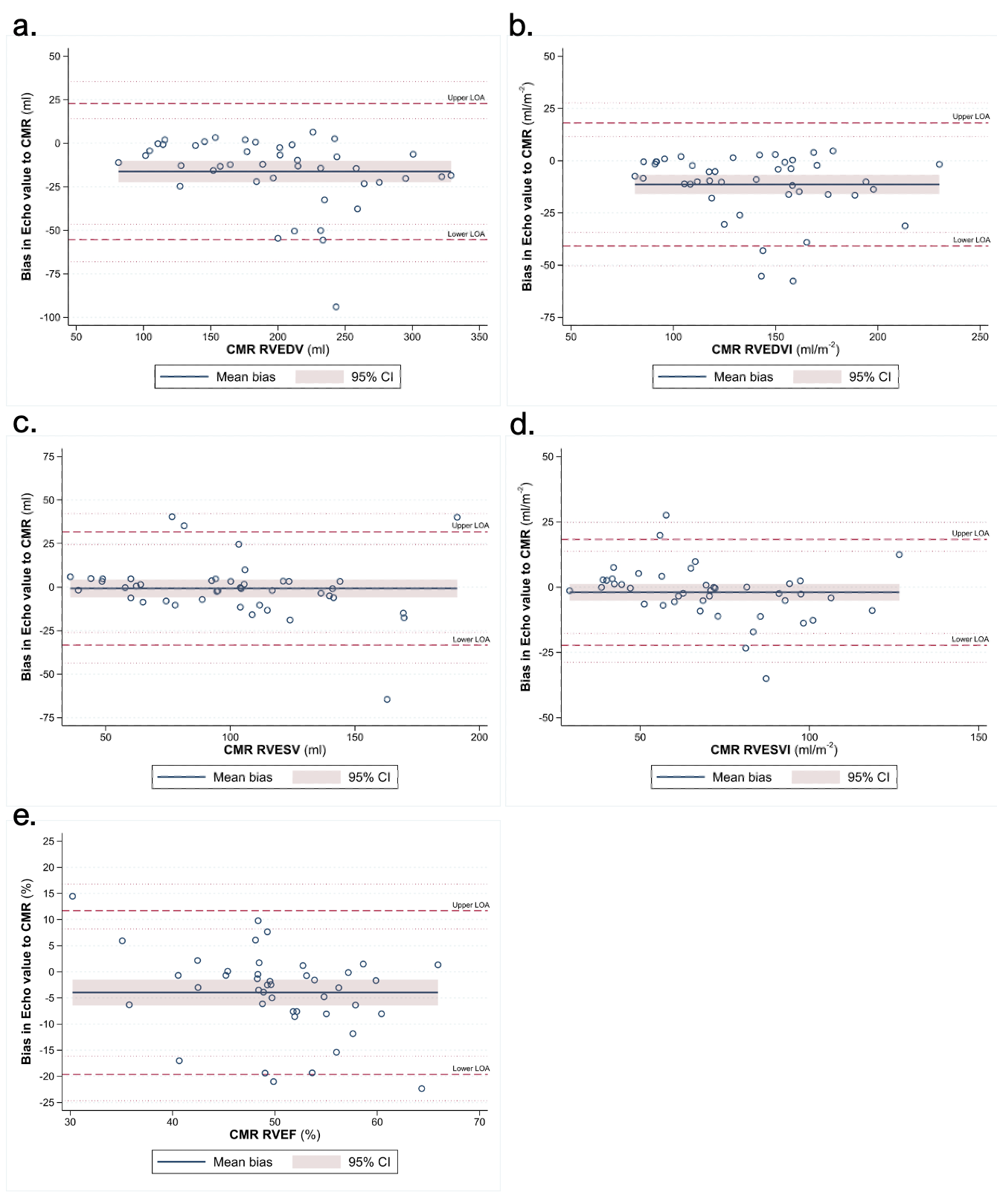

Supplement: S1 Fig — In each panel, the y-axis shows the bias (3D Echo – CMR) plotted against the corresponding CMR measurement on the x-axis. The solid blue line is the mean bias; the pink shaded band represents its 95% confidence interval (CI). Red dashed lines indicate the limits of agreement (LOA), and red dotted lines indicate the 95% CI around each LOA. (a): RV end-diastolic volume (RVEDV). (b): RVEDV indexed to body surface area (RVEDVi). (c): RV end-systolic volume (RVESV). (d): RVESV indexed to body surface area (RVESVi). (e): RV ejection fraction (RVEF). (PNG) (Add Supplymentary Fig) [file pone.0344373.s001.png]
