# Supplementary material for: Utility of three-dimensional echocardiography for evaluating right ventricular size and function and ventricular myocardial deformation in repaired tetralogy of fallot
Source: PLoS One. 2026 Mar 17;21(3):e0344373. doi: 10.1371/journal.pone.0344373 (PMC12994780; doi:10.1371/journal.pone.0344373)
Supplement: Attachment — Submitted filename: KS 13.2.2026 Response to reviewer_PW19jan_2.docx [file pone.0344373.s003.docx]

**Utility of Three-Dimensional Echocardiography for Evaluating Right Ventricular Size and Function and Ventricular Myocardial Deformation in Repaired Tetralogy of Fallot**

Thank you very much for your kind considering on our manuscript. We appreciate the constructive and very helpful feedback and the supportive comments from the reviewers. We have carefully considered the reviewers’ comments and suggestions and have edited the manuscript to address them. We believe the manuscript has been greatly improved after the revision based on those suggestions.

We have addressed each comment as outlined below with the yellow color highlight in the manuscript

When submitting your revision, we need you to address these additional requirements.

1.Please ensure that your manuscript meets PLOS ONE's style requirements, including those for file naming.

**Answer:** Our manuscript meets PLOS ONE's style requirements

2. Please note that funding information should not appear in any section or other areas of your manuscript. We will only publish funding information present in the Funding Statement section of the online submission form. Please remove any funding-related text from the manuscript.

**Answer:** Remove funding-related text from the manuscript.

3. We note that you have indicated that there are restrictions to data sharing for this study. PLOS only allows data to be available upon request if there are legal or ethical restrictions on sharing data publicly. For more information on unacceptable data access restrictions, please see http://journals.plos.org/plosone/s/data-availability#loc-unacceptable-data-access-restrictions.

Before we proceed with your manuscript, please address the following prompts:

a) If there are ethical or legal restrictions on sharing a de-identified data set, please explain them in detail (e.g., data contain potentially identifying or sensitive patient information, data are owned by a third-party organization, etc.) and who has imposed them (e.g., a Research Ethics Committee or Institutional Review Board, etc.). Please also provide contact information for a data access committee, ethics committee, or other institutional body to which data requests may be sent.

b) If there are no restrictions, please upload the minimal anonymized data set necessary to replicate your study findings to a stable, public repository and provide us with the relevant URLs, DOIs, or accession numbers. For a list of recommended repositories, please see

https://journals.plos.org/plosone/s/recommended-repositories. You also have the option of uploading the data as Supporting Information files, but we would recommend depositing data directly to a data repository if possible.

We will update your Data Availability statement on your behalf to reflect the information you provide.

**Answer:** The data underly­ing this study cannot be made publicly available due to patient confidentiality restrictions. However, the data will be made available to qualified researchers upon reasonable request. Data requests may be directed to the Research Ethics Committee of the Faculty of Medicine, Chiang Mai University which is responsible for oversight of data governance and access. Please contact: Research Ethics Committee, Faculty of Medicine, Chiang Mai University Address: Rianroum Buiding, 3rd floor Faculty of Medicine, Chiang Mai University 110 Intavaroros Road, Tambon Sriphum, Amphoe Muang, Chiang Mai Thailand 50200 Business Tel.: No. +6653936643, +6653936641, +6653935279 ext. 22-23 Email: research.med.cmu@gmail.com, researchmed@cmu.ac.th. This committee is independent of the study authors and is authorized to review and approve data access requests in accordance with insti­tutional policies and ethical guidelines.

4. Your ethics statement should only appear in the Methods section of your manuscript. If your ethics statement is written in any section besides the Methods, please delete it from any other section.

**Answer:** Ethics statement only appears in the Methods section of your manuscript.

5. We note that Figure 1 in your submission contain copyrighted images. All PLOS content is published under the Creative Commons Attribution License (CC BY 4.0), which means that the manuscript, images, and Supporting Information files will be freely available online, and any third party is permitted to access, download, copy, distribute, and use these materials in any way, even commercially, with proper attribution. For more information, see our copyright guidelines: http://journals.plos.org/plosone/s/licenses-and-copyright.

 We require you to either (1) present written permission from the copyright holder to publish these figures specifically under the CC BY 4.0 license, or (2) remove the figures from your submission:

 1. You may seek permission from the original copyright holder of Figure 1 to publish the content specifically under the CC BY 4.0 license.

 We recommend that you contact the original copyright holder with the Content Permission Form (http://journals.plos.org/plosone/s/file?id=7c09/content-permission-form.pdf) and the following text:

“I request permission for the open-access journal PLOS ONE to publish XXX under the Creative Commons Attribution License (CCAL) CC BY 4.0 (http://creativecommons.org/licenses/by/4.0/). Please be aware that this license allows unrestricted use and distribution, even commercially, by third parties. Please reply and provide explicit written permission to publish XXX under a CC BY license and complete the attached form.”

 Please upload the completed Content Permission Form or other proof of granted permissions as an "Other" file with your submission.

 In the figure caption of the copyrighted figure, please include the following text: “Reprinted from [ref] under a CC BY license, with permission from [name of publisher], original copyright [original copyright year].”

 2. If you are unable to obtain permission from the original copyright holder to publish these figures under the CC BY 4.0 license or if the copyright holder’s requirements are incompatible with the CC BY 4.0 license, please either i) remove the figure or ii) supply a replacement figure that complies with the CC BY 4.0 license. Please check copyright information on all replacement figures and update the figure caption with source information. If applicable, please specify in the figure caption text when a figure is similar but not identical to the original image and is therefore for illustrative purposes only.

**Answer:** Thank you very much for your careful review and for raising this important copyright consideration. We would like to clarify that **all echocardiography and cardiac MRI images presented in Figure 1 are original images generated by the authors from our own patient data**, acquired and analyzed at our institution as part of routine clinical care and this research study.

We sincerely apologize for the misunderstanding. We understand that the concern relates specifically to the clip art elements used in the original Figure 1 (including the crowd illustration and other artistic components)

In response to this concern, we have completely removed the previous version of Figure 1 and have created a new figure to replace it. The revised Figure 1 has been fully reconstructed by the authors using original schematic designs, without incorporating any third-party clip art, stock images, or copyrighted artistic materials.

The new figure is entirely author-generated and does not contain any copyrighted visual elements from external sources. Therefore, there are no copyright restrictions associated with the revised figure.

We appreciate the journal office’s careful review and thank you for giving us the opportunity to correct this issue.

6. If the reviewer comments include a recommendation to cite specific previously published works, please review and evaluate these publications to determine whether they are relevant and should be cited. There is no requirement to cite these works unless the editor has indicated otherwise. 
**Answer:** We appreciate the reviewer’s thoughtful suggestions, which helped us reassess and strengthen the contextual foundation of our work.

**Additional Editor Comments:**

I would like to thank the authors for their very interesting and clinically relevant research work. In addition to addressing the comments raised by the reviewers, I would like to request the authors to address the following issues. Particularly, I would like to invite the authors special attention related to the methods and conclusion section of the study since there are major issues raised by the reviewers.

- Line 145-146: “The cardiac function was evaluated by a single experienced imaging pediatric cardiologist (KS).” I assume that KS is the first author of this manuscript. If that is the case, isn’t there an observer bias? How about the impact of using single assessor on the impact of the accuracy of the data set?

**Answer:** Thank you for this important and thoughtful comment. Yes, KS is the first author of this manuscript and served as the primary imaging cardiologist for cardiac magnetic resonance (CMR) analysis. We acknowledge that the use of a single assessor may raise concerns regarding potential observer bias. However, this approach reflects the **real-world clinical practice and resource limitations in Northern Thailand,** where there is currently **only one pediatric cardiologist with specialized expertise in congenital cardiac MRI interpretation.** In this setting, the use of a single assessor is unavoidable and represents standard clinical practice. Importantly, KS holds **Society for Cardiovascular Magnetic Resonance (SCMR) Level III certification,** which represents the highest level of formal training and competency in CMR acquisition, interpretation, and reporting. This level of expertise helps ensure high-quality and reliable measurements. To further minimize potential observer bias, all CMR analyses were performed using **standardized, validated post-processing protocols,** and the assessor was **blinded to echocardiographic strain results and relevant clinical data** at the time of analysis. In addition, measurements were obtained using **semi-automated software with predefined contouring algorithms,** reducing subjective influence on volumetric assessment. We acknowledge that the lack of inter-observer variability analysis is a limitation of the study, and this has now been explicitly addressed in the **Study Limitations** section.

“Several limitations of this study should be acknowledged. First, the relatively modest sample size may limit the generalizability of our findings and precluded robust subgroup analyses, including stratification by surgical strategy or duration since definitive repair. Second, image quality remains a challenge for 3DE, particularly in patients with suboptimal acoustic windows or marked right ventricular dilation. In such cases, incomplete right ventricular coverage within a single acquisition may lead to systematic underestimation of volumetric and functional parameters, reducing measurement reliability. Consequently, patients with very advanced right ventricular remodeling, significant residual shunts, or arrhythmias were excluded, and our findings may not be directly applicable to these clinically important subgroups, for whom CMR imaging remains the reference standard for accurate assessment of right ventricular size and systolic function. Third, echocardiographic strain measurements may be influenced by vendor-specific analysis algorithms, which could affect reproducibility across platforms. In addition, the use of a single experienced assessor may raise concerns regarding potential observer bias, although standardized post-processing protocols and blinding between echocardiographic and CMR analyses were applied to mitigate this risk. Furthermore, only moderate correlations were observed between 2D and 3D strain parameters, highlighting modality-specific differences and underscoring the importance of interpreting strain values within the context of the imaging technique used. Despite these limitations, the study reflects real-world clinical practice and benefits from standardized same-day echocardiography and CMR acquisition, minimizing physiologic variability. Future larger, multicenter studies with multiple independent readers are warranted to validate the reproducibility of 3DE-derived parameters and to further define their role in risk stratification and long-term outcome assessment in patients with rTOF”.

**Study limitation**

**Pages 18 and 19; lines 349-371**

Page: 16, lines 312-314

- **Lines 168- 172: what was the basis to determine sample size by taking the mean bias of 5 and SD of 8? Do you think these values help to produce a stable model? Needs elaboration!**

**Answer:** We thank the reviewer for raising this important point. The justification for the assumed effect size of the mean bias and its variance was based on findings from our pilot study [1]. In addition, we accounted for within-subject correlation by assuming an intra-subject correlation coefficient of 0.5.

As our primary objective was to compare the mean right ventricular ejection fraction (RVEF) between echocardiography and cardiac magnetic resonance (CMR), we estimated the mean bias as the difference in mean RVEF obtained from the two modalities. Our intention was not to derive a precise or stable estimate, but rather to explore and describe patterns of disagreement between the measurement methods. Accordingly, residual and systematic differences were evaluated using the Bland Altman approach. Because our analysis was not based on a formal statistical parametric model, the sample size calculation derived from this approach should not lead to the concern raised by the reviewer

Reference

1. Shieh, G. (2013). On Using a Pilot Sample Variance for Sample Size Determination in the Detection of Differences between Two Means: Power Consideration. *Psicologica, 34*, 125-143.

- **Line 168: sample size calculated using pilot study. Why sample was not calculated based on previous study findings?**

**Answer:** We appreciate the reviewer’s comment. Sample size estimation is typically used to determine the acceptable statistical power for hypothesis testing based on an expected effect size. In the present study, we chose to base the effect size on our own pilot data, as we considered our study population to best reflect the variance and effect size in our target population [1], rather than relying on assumptions from other populations with potentially different characteristics.In addition, there is limited published evidence describing the mean bias of RVEF between echocardiography and CMR in patients with tetralogy of Fallot (TOF).

Reference

1. Shieh, G. (2013). On Using a Pilot Sample Variance for Sample Size Determination in the Detection of Differences between Two Means: Power Consideration. *Psicologica, 34*, 125-143.

- **Lines 171- 172: States that “calculations were performed using a two sampled-paired t-test”.  However, comparison of RVEF by 3DE vs CMR is agreement comparison, not a difference in means comparison. Please explain this.**

**Answer:** We appreciate the reviewer’s feedback and agree that mean bias alone does not fully capture agreement, as it reflects only the systematic difference between the two measurement methods [1]. Agreement between the modalities was therefore assessed using the Bland Altman method, which evaluates both systematic and residual differences. The calculation of the mean bias and its 95% confidence interval forms an integral part of the Bland Altman analysis [2]. We additionally included hypothesis testing to inform whether the observed systematic bias was statistically significant.

References

1. Bland, J. M., and D. G. Altman. 1986. Statistical methods for assessing agreement between two methods of clinical measurement. Lancet 327: 307–310. <https://doi.org/10.1016/S0140-6736(86)90837-8>.
2. Mokkink, L.B., Boers, M., van der Vleuten, C.P.M. *et al.* COSMIN Risk of Bias tool to assess the quality of studies on reliability or measurement error of outcome measurement instruments: a Delphi study. *BMC Med Res Methodol* **20**, 293 (2020). https://doi.org/10.1186/s12874-020-01179-5

- Lines 162- 163: What is the importance of using LOA alongside P10? Do the two parameters convey different info?

**Answer:** The limits of agreement (LOA) inform us about the width of the 95% error range observed in the data. In contrast, *P10* represents the proportion of measurements that fall within a 10% error margin from the reference standard, which in this study is CMR. Similar to laboratory measurement standards, acceptable error thresholds are often predefined, with 15% commonly considered acceptable. While the Bland Altman LOA assume a normal distribution of differences across the measurement range, *P10* provides complementary information by summarizing overall accuracy, irrespective of the magnitude of individual values.

- Lines 186-192:
  - Mean bias is reported, but 95% limits of agreement are missing.

**Answer:** Thank you for your suggestion. We have added the 95% limits of agreement to the text in accordance with your recommendation.

- RVESVi correlation is stated as interval (r = 0.90–0.88) and its bias is described as “minimal”. What does “minimal bias” means?

**Answer:** We thank the reviewer for this helpful suggestion. As the degree of disagreement was not yet purposed in this parameter, we have removed this term to ensure the Results section is more precise and focused.

- Lines 196- 204: Please, explain the clinical relevance of such a small difference between the 2D and 3D GLS of RV strain values though the two have a statistically significant difference.

**Answer:** We agree that the absolute difference between 2D- and 3D-derived right ventricular (RV) global longitudinal strain (GLS) values is relatively small, despite being statistically significant. Therefore, this numerical difference alone should not be overinterpreted in isolation.However, the **clinical relevance of three-dimensional echocardiography (3DE)** extends beyond the modest difference in GLS values. A key advantage of 3DE is its ability to provide a **comprehensive assessment of right ventricular size and systolic function,** which demonstrated **strong correlation with cardiac magnetic resonance (CMR), the reference gold standard**, in our study. This capability is not achievable with conventional 2D strain analysis. Importantly, 3DE also allows for **simultaneous assessment of RV myocardial deformation using 3D strain analysis.** In our study, RV strain parameters derived from both 2D and 3D echocardiography were significantly impaired in patients with **CMR-defined RV systolic dysfunction,** supporting the physiological relevance of strain abnormalities. Notably, 3D RV GLS showed superior discriminatory performance compared with 2D RV GLS for identifying RV systolic dysfunction. While 2D RV GLS is easier to acquire and widely available, it provides **only deformation analysis without direct volumetric or functional assessment of the right ventricle**. In contrast, 3DE offers an integrated evaluation of **RV volume, ejection fraction, and myocardial strain within a single imaging modality,** making it particularly valuable for longitudinal follow-up and clinical decision-making in patients with repaired Tetralogy of Fallot.We have clarified this point in the revised manuscript to emphasize that the added value of 3DE lies in its **comprehensive functional assessment**, rather than the small absolute difference in GLS values alone. We appreciate the reviewer’s comment, which helped us strengthen the clinical interpretation of our findings.

“The major clinical advantage of three-dimensional echocardiography lies not in the small numerical difference in strain values, but in its ability to provide a comprehensive, integrated assessment of right ventricular size, systolic function, and myocardial deformation within a single imaging modality”.

**Discussion- Clinical Implication**

**Page 17, lines 342-344**

- Lines 232- 235: it appears an overstated claim.

**Answer:** Thank you for this comment. We agree that the statement in Lines 232–235 may have appeared overstated, particularly regarding the novelty and scope of the study. To address this concern, we have **revised the wording to adopt a more cautious and balanced tone,** avoiding definitive or exclusive claims while maintaining the scientific contribution of the work. Specifically, we have removed language suggesting priority or uniqueness and instead emphasized that our study **adds to the existing literature** by providing a comprehensive comparison of 2D and 3D echocardiographic parameters with CMR in patients with repaired Tetralogy of Fallot. We appreciate the reviewer’s suggestion, which has helped improve the clarity and appropriateness of the manuscript’s interpretation.

“This study demonstrates the clinical utility of three-dimensional echocardiography (3DE) for the assessment of right ventricular size, function, and myocardial deformation in patients with repaired Tetralogy of Fallot. Our findings contribute to the existing body of evidence by comparing two-dimensional and three-dimensional echocardiographic parameters with cardiac magnetic resonance–derived right ventricular function, supporting the complementary role of 3DE in the evaluation of right ventricular performance”.

**Discussion**

**Page 14, Lines 263-267**

- Lines 237- 252: The discussion mentions the “strong correlation” between 3DE and MRI as an indicator of agreement between the two diagnostic modalities. However, correlation indicates association not agreement between two diagnostic modalities. Please check and revise accordingly as it may mislead readers.

**Answer:** Thank you for this important methodological clarification. We agree with the reviewer that **correlation is not reflects agreement,** and that using correlation alone to infer agreement between diagnostic modalities may be misleading. To address this concern, we have **revised the Discussion to clearly distinguish between correlation and agreement**, and to emphasize that agreement was evaluated using **Bland-Altman analysis and measurement bias,** rather than correlation coefficients alone. Specifically, we now state that while strong correlations were observed between 3DE and CMR for RV volumetric parameters, **agreement was supported by modest mean bias and acceptable limits of agreement,** consistent with prior validation studies. The revised text avoids equating correlation with agreement and more accurately reflects the statistical methods used. We appreciate the reviewer’s comment, which helped improve the methodological rigor and clarity of the manuscript.

- In our cohort, 3DE demonstrated a strong association with CMR for right ventricular volumetric measurements. While correlation analysis showed a strong linear relationship between 3DE- and CMR-derived RV volumes, agreement between the two modalities was more appropriately assessed using Bland–Altman analysis. This analysis demonstrated a modest mean bias with acceptable limits of agreement, supporting the clinical feasibility of 3DE for RV volumetric assessment in routine practice
- Despite these discrepancies, the magnitude of underestimation was small and remained within clinically acceptable limits for longitudinal follow-up in patients with rTOF. Page

**Discussion**

**Page 14, Lines 269-274, 277-279**

- Lines 256- 259: The authors stated that “*Our study demonstrated that 3DE RV GLS has a moderate correlation with 2DE GLS. Three-DE RV GLS had significantly more negative values than 2DE-derived RV-GLS. This suggests that 3D RV GLS imaging may provide a more sensitive detection of RV myocardial deformation compared to 2D assessment..”.* It may be misleading to infer that the 3DE RV GLS has more negative values than 2DE RV-GLS and that implies better performance of 3DE RV GLS. Please, check it and revise accordingly.

**Answer:** Thank you for this important comment. We agree with the reviewer that stating that 3DE RV GLS values are more negative than 2DE RV GLS could be misinterpreted as implying superior performance of 3DE. This was not our intention. The observed difference primarily reflects **methodological differences between two-dimensional and three-dimensional speckle-tracking techniques**, rather than an inherent superiority of one modality over the other. To address this concern, we have **revised the Discussion to clarify that the difference in GLS values should not be interpreted as improved performance or higher sensitivity**, but rather as a consequence of differences in tracking algorithms and the ability of 3DE to incorporate out-of-plane myocardial motion. We now emphasize that **strain values are modality-specific and should not be used interchangeably across techniques**, and that clinical interpretation should rely on modality-consistent reference values and diagnostic performance metrics. We appreciate the reviewer’s comment, which helped us improve the accuracy and clarity of our interpretation.

“In our study, 3DE derived RV GLS demonstrated a moderate association with 2D RV GLS. The systematically more negative GLS values observed with 3DE reflect **methodological differences between 2D and 3D speckle-tracking approaches**, including the incorporation of out-of-plane myocardial motion in 3DE, rather than superior performance of one technique over the other. Therefore, RV GLS values should be interpreted in a **modality-specific manner** and should not be directly compared across imaging techniques”.

**Discussion**

**Page 15, line 288-293**

- **Lines 277- 283: The authors attributed the lack of statistical significance to the “small sample size”. However, you also claimed that the study was adequately powered (power 90%). Hence, the two ideas seem to contradict.**

**Answer:** We apologise for the confusion in the manuscript. The small sample sizes referred to were specific to the comparison of ROC curves, for which our sample size estimation was not designed to address this hypothesis. Consequently, the lack of statistical significance may be attributable to either a small effect size or limited sample size. We have revised the sentence to provide a more accurate interpretation, in lines 307-314 as follows:

“In our study, the highest diagnostic performance was observed when combining RVEF with both 2D and 3D RV-GLS, achieving an AUC of 0.93 (95% CI: 0.83–0.99). These findings underscore the superior discriminative ability of 3D RV-GLS over conventional volumetric indices and 2D strain alone. This is likely attributed to the ability of 3DE to capture the complex RV geometry more accurately, thereby allowing for a comprehensive analysis of myocardial deformation. Although a clear trend was observed, indicating the added diagnostic value of 3D RV-GLS, there is no statistical significance.”

**And in limitation section in lines 350-354 as follows:**

**“**First, our sample size may be relatively small for some secondary objectives of this study. In addition, multiple hypothesis testing was performed, which increases the possibility that some statistically significant findings may have occurred by chance. The focus study to confirm those findings in our study is warranted.”

**Discussion**

**Page 16, lines 307-314**

**Limitation, lines 350-354**

- **Lines 305- 316: The limitations of the study mentioned under this paragraph appear to be general rather than specific to their study. Here are the reasons: small sample size is mentioned as limitation while you already told us in the methods section that the study was adequately powered; image quality is mentioned as limitation for 3DE while you told us that you have excluded cases that had poor image quality; result differences based on vendor specific strain analysis is mentioned as limitation though that was not related to the current study. Please clarify or revise accordingly.**

**Answer:** We agree that patients with markedly dilated right ventricles, residual shunts, and arrhythmias represent a clinically important subgroup, particularly with respect to decision-making for interventions such as pulmonary valve replacement. However, these exclusion criteria were applied to ensure technical validity and measurement reliability, as suboptimal three-dimensional echocardiographic image quality especially incomplete RV coverage in severely dilated ventricles can lead to inaccurate volumetric and strain measurements. We have clarified this rationale in the Study Limitations section and acknowledge that our findings may not be directly generalizable to patients with very advanced RV remodeling or complex residual lesions. In this context, cardiac magnetic resonance imaging remains the reference standard for accurate evaluation of right ventricular size and systolic function, particularly in patients with advanced disease or complex anatomy. **Nevertheless, our findings provide clinically relevant insights into the performance of three-dimensional echocardiography in a well-defined and commonly encountered rTOF population, supporting its role as a complementary modality for routine follow-up.** Regarding the inclusion of patients who had undergone palliative procedures, we would like to clarify that all patients in this cohort had a final anatomical diagnosis of repaired Tetralogy of Fallot, and the term “palliated” refers to patients who underwent initial staged palliation prior to definitive surgical repair. These patients subsequently received complete repair and were included in the analysis as part of the repaired TOF population. We agree that staged and primary repair strategies may influence RV loading conditions and myocardial mechanics; however, the present study was not powered to perform subgroup analyses based on surgical strategy. This has now been acknowledged as a limitation. **Despite this, the inclusion of patients with varied surgical pathways reflects real-world clinical practice and enhances the external relevance of our findings within the repaired TOF population.** We also agree that the interval between definitive repair and imaging may influence RV size, function, and strain patterns. Although the duration since repair was recorded, the relatively modest sample size limited our ability to perform robust correlation or subgroup analyses based on time since repair. We have now explicitly addressed this point in the Study Limitations sections and suggest that future larger, longitudinal studies are needed to evaluate the impact of surgical timing and repair strategy on RV remodeling and functional parameters. **Nonetheless, the standardized same-day imaging protocol in our study minimizes physiologic variability and strengthens the internal validity of the observed associations.** We appreciate the reviewer’s insightful comments, which have helped us better contextualize the applicability and limitations of our findings in clinical practice

#### “**Study Limitations**

Several limitations of this study should be acknowledged. First, the relatively modest sample size may limit the generalizability of our findings and precluded robust subgroup analyses, including stratification by surgical strategy or duration since definitive repair. Second, image quality remains a challenge for 3DE, particularly in patients with suboptimal acoustic windows or marked right ventricular dilation. In such cases, incomplete right ventricular coverage within a single acquisition may lead to systematic underestimation of volumetric and functional parameters, reducing measurement reliability. Consequently, patients with very advanced right ventricular remodeling, significant residual shunts, or arrhythmias were excluded, and our findings may not be directly applicable to these clinically important subgroups, for whom CMR imaging remains the reference standard for accurate assessment of right ventricular size and systolic function. Third, echocardiographic strain measurements may be influenced by vendor-specific analysis algorithms, which could affect reproducibility across platforms. In addition, the use of a single experienced assessor may raise concerns regarding potential observer bias, although standardized post-processing protocols and blinding between echocardiographic and CMR analyses were applied to mitigate this risk. Furthermore, only moderate correlations were observed between 2D and 3D strain parameters, highlighting modality-specific differences and underscoring the importance of interpreting strain values within the context of the imaging technique used. Despite these limitations, the study reflects real-world clinical practice and benefits from standardized same-day echocardiography and CMR acquisition, minimizing physiologic variability. Future larger, multicenter studies with multiple independent readers are warranted to validate the reproducibility of 3DE-derived parameters and to further define their role in risk stratification and long-term outcome assessment in patients with rTOF”.

**Study Limitations**

**Pages 18 and 19, Line 249-373**

- **Does good AUC imply good agreement between 3DE and CMR and vise-versa? Also, the CMR is both used as gold standard and comparator. Give us short explanation in the discussion.**

**Answer:** Thank you for your suggestion. A high AUC indicates the ability of a parameter or tool to discriminate an outcome, in this case, reduced right ventricular ejection fraction (RVEF < 48%), rather than agreement between measurement methods.

As CMR is not widely available, we aimed to explore whether parameters derived from 3DE could serve as predictors for identifying patients with reduced right ventricular function. Our findings suggest that 3DE parameters may have potential utility as a screening tool for surveillance of patients with repaired tetralogy of Fallot (rTOF) and may assist clinicians in selecting patients who would benefit from further evaluation with CMR. We have added a brief explanation in the manuscript accordingly, as follows:

“When stratified by RVEF ≥48% vs. <48%, patients with reduced RVEF exhibited significantly less negative strain values across both 2D and 3D modalities, supporting the role of myocardial strain as an early marker of RV dysfunction (8, 15, 29). A high AUC indicates the ability of a parameter or tool to discriminate patient who have or doesn’t have RVEF < 48%. Notably, 3DE-derived RV-GLS exhibited a superior trend of AUC for identifying this outcome. compared to 2DE. This suggests that 3DE-derived strain analysis potentially serve as a non-invasive tool for early identification of ventricular dysfunction in rTOF patients, potentially reducing the need for frequent CMR evaluations (13).”

**Discussion**

**Lines 298-303**

Reviewers' comments:

Reviewer's Responses to Questions

**Comments to the Author**

1. Is the manuscript technically sound, and do the data support the conclusions?

The manuscript must describe a technically sound piece of scientific research with data that supports the conclusions. Experiments must have been conducted rigorously, with appropriate controls, replication, and sample sizes. The conclusions must be drawn appropriately based on the data presented.

Reviewer #1: Partly

Reviewer #2: Yes

**Answer:** Following reviewer feedback, the **Conclusion section has been revised to adopt a more conservative and balanced interpretation,** ensuring that the conclusions are fully supported by the data presented. The revised conclusions emphasize the **complementary and supportive role of three-dimensional echocardiography,** rather than overstating its clinical impact or implying equivalence to CMR.

#### ****Conclusion****

Three-dimensional echocardiography demonstrates a consistent association with CMR for the assessment of right ventricular volumes but modest underestimation of volumetric and functional parameters. Abnormal right ventricular strain was observed in patients with CMR-defined systolic dysfunction, supporting the clinical relevance of strain analysis. Overall, 3DE may serve as a feasible complementary tool for longitudinal right ventricular assessment in patients with rTOF, alongside CMR as the reference standard.

**Page 19, lines 373-378**

2. Has the statistical analysis been performed appropriately and rigorously?

Reviewer #1: Yes

Reviewer #2: Yes

3. Have the authors made all data underlying the findings in their manuscript fully available?

The [PLOS Data policy](http://track.editorialmanager.com/CL0/http:%2F%2Fwww.plosone.org%2Fstatic%2Fpolicies.action%23sharing/1/010f019bb203d5af-7764a7ea-c740-471f-bfbe-4f7befac29fd-000000/UsFYha4Bgib2sSs4i9eLVFqLW1RANKEnzRKRUsK9xi0=245) requires authors to make all data underlying the findings described in their manuscript fully available without restriction, with rare exception (please refer to the Data Availability Statement in the manuscript PDF file). The data should be provided as part of the manuscript or its supporting information, or deposited to a public repository. For example, in addition to summary statistics, the data points behind means, medians and variance measures should be available. If there are restrictions on publicly sharing data—e.g. participant privacy or use of data from a third party—those must be specified.

Reviewer #1: Yes

Reviewer #2: No

4. Is the manuscript presented in an intelligible fashion and written in standard English?

PLOS ONE does not copyedit accepted manuscripts, so the language in submitted articles must be clear, correct, and unambiguous. Any typographical or grammatical errors should be corrected at revision, so please note any specific errors here.

Reviewer #1: Yes

Reviewer #2: Yes

5. Review Comments to the Author

Please use the space provided to explain your answers to the questions above. You may also include additional comments for the author, including concerns about dual publication, research ethics, or publication ethics. (Please upload your review as an attachment if it exceeds 20,000 characters)

Reviewer #1: Thank you for the opportunity to review the paper.
1. My general comments about the research work
The research tries to address a clinically relevant and challenging long-term issue in patients who had undergone surgical repair for Tetralogy of Fallot (both staged and primary repair).The study design is appropriate with detailed imaging protocols and a gold standard (CMR). The attempt to minimize physiologic variability impacts by doing the imaging studies on the same day is another strength of the study. However, in view of the modest correlation observed between 3D ECHO and CMR in quantification of right ventricular systolic function, the clinical implication of the research findings is slightly overstated.

**Answer:** We sincerely thank the reviewer for the thoughtful and constructive overall evaluation of our work and for recognizing the clinical relevance of the research question, the appropriate study design, the detailed imaging protocols, and the strength of performing echocardiography and CMR on the same day to minimize physiologic variability.

We agree with the reviewer that, given the **modest correlation observed between 3D echocardiography–derived and CMR-derived right ventricular systolic function**, the clinical implications of our findings should be interpreted with appropriate caution. In response to this comment, we have **revised the Discussion and Conclusion sections to adopt a more conservative and balanced tone**, avoiding any implication that 3D echocardiography can replace CMR for precise quantification of RV systolic function.

Specifically, we now emphasize that **CMR remains the reference standard**, and that the value of 3D echocardiography lies in its **complementary role** particularly for longitudinal surveillance and functional trend assessment in routine clinical practice rather than as a definitive alternative. The revised text clarifies that our findings support the **feasibility and supportive clinical utility** of 3D echocardiography, rather than overstat ing its diagnostic equivalence to CMR.

We appreciate the reviewer’s insightful comment, which has helped us refine the interpretation and clinical framing of our results.

**Discussion**

In our cohort, 3DE demonstrated a strong association with cardiac magnetic resonance (CMR) for right ventricular volumetric measurements. While correlation analysis showed a strong linear relationship between 3DE- and CMR-derived RV volumes, agreement between the two modalities was more appropriately assessed using Bland–Altman analysis. This analysis demonstrated a modest mean bias with acceptable limits of agreement, supporting the clinical feasibility of 3DE for RV volumetric assessment in routine practice

Nonetheless, the moderate correlation observed indicates the potential for 3DE to be useful for identifying patients with clinically relevant RV dysfunction, especially in settings where CMR availability is limited.

In our study, 3DE derived RV GLS demonstrated a moderate association with 2D RV GLS. The systematically more negative GLS values observed with 3DE reflect **methodological differences between 2D and 3D speckle-tracking approaches**, including the incorporation of out-of-plane myocardial motion in 3DE, rather than superior performance of one technique over the other. Therefore, RV GLS values should be interpreted in a **modality-specific manner** and should not be directly compared across imaging techniques

#### ****Conclusion****

Three-dimensional echocardiography demonstrates a consistent association with CMR for the assessment of right ventricular volumes but modest underestimation of volumetric and functional parameters. Abnormal right ventricular strain was observed in patients with CMR-defined systolic dysfunction, supporting the clinical relevance of strain analysis. Overall, 3DE may serve as a feasible complementary tool for longitudinal right ventricular assessment in patients with rTOF, alongside CMR as the reference standard.

**Discussion**

**Page 14, lines 269-274**

**Page 14, lines 277-279**

**Pages 15, lines 288-293**

**Conclusion: page 3, lines 45-51; pages 19, lines 372-378**

2. Specific comments for improvement
• Methods section: whether the 2D/3D echocardiographer is blinded from the CMR findings and vise versa is unclear to avoid bias.

**Answer:** Thank you for this important comment regarding potential observer bias.

Although two-dimensional (2D) and three-dimensional (3D) echocardiography and cardiac magnetic resonance (CMR) were performed on the same day to minimize physiologic variability, **all post-processing analyses were conducted on separate days.** Importantly, the echocardiographic analyses including **2D strain analysis and 3D RV volume and strain measurements** were performed by an investigator who was **blinded to the CMR findings** at the time of analysis.

Similarly, CMR post-processing and interpretation were performed independently and **without knowledge of the echocardiographic results**. This blinding strategy was implemented to minimize potential observer bias and ensure objective assessment across imaging modalities.

We have clarified this point in the **Methods section** of the revised manuscript.

“**Blinding and Image Analysis**

Two-dimensional (2D) and 3DE and CMR were performed on the same day to minimize physiologic variability. However, all post-processing analyses were conducted on separate days. Echocardiographic analyses, including 2D right ventricular strain and 3D right ventricular volumetric and strain measurements, were performed by an investigator who was blinded to the CMR results at the time of analysis. Likewise, CMR post-processing and interpretation were performed independently and without knowledge of the echocardiographic findings. This blinding strategy was implemented to minimize observer bias and ensure objective comparison between imaging modalities”.

**Method**

**Pages 8 and 9, lines 155-163**

• Discussion: The study fails to sufficiently explain/discuss the discrepancy between the 3D ECHO and CMR with respect to RV systolic function assessment. Due to the modest correlation between 3D ECHO and CMR in RV systolic function assessment in the study, the first statement on clinical implications appear to be slightly overstated.

**Answer:** Thank you for this important comment.

We agree that the discrepancy between three-dimensional echocardiography (3DE) and cardiac magnetic resonance (CMR) in the assessment of right ventricular (RV) systolic function required further clarification. To address this, we have **expanded the Discussion section** to more explicitly explain the observed differences between modalities. The revised text now discusses several contributing factors, including differences in spatial resolution, dependence on acoustic windows, challenges in endocardial border delineation in a dilated and complex RV geometry, and the impact of foreshortening and temporal resolution on 3DE-derived RV ejection fraction. These factors help explain the modest correlation observed between 3DE and CMR for RV systolic function.

In addition, we agree that the initial statement regarding clinical implications may have appeared overstated in light of the modest correlation. Accordingly, we have **revised the Discussion and Conclusion sections to adopt a more conservative and balanced interpretation.** The revised text emphasizes that **CMR remains the reference standard for RV systolic function assessment,** and that the role of 3DE should be considered **complementary,** particularly for longitudinal follow-up and functional trend assessment, rather than as a replacement for CMR.

We appreciate the reviewer’s comment, which has helped improve both the methodological discussion and the clinical framing of our findings.

**Discussion**

In our cohort, 3DE demonstrated a strong association with cardiac magnetic resonance (CMR) for right ventricular volumetric measurements. While correlation analysis showed a strong linear relationship between 3DE- and CMR-derived RV volumes, agreement between the two modalities was more appropriately assessed using Bland–Altman analysis. This analysis demonstrated a modest mean bias with acceptable limits of agreement, supporting the clinical feasibility of 3DE for RV volumetric assessment in routine practice

Nonetheless, the moderate correlation observed indicates the potential for 3DE to be useful for identifying patients with clinically relevant RV dysfunction, especially in settings where CMR availability is limited.

In our study, 3DE derived RV GLS demonstrated a moderate association with 2D RV GLS. The systematically more negative GLS values observed with 3DE reflect **methodological differences between 2D and 3D speckle-tracking approaches**, including the incorporation of out-of-plane myocardial motion in 3DE, rather than superior performance of one technique over the other. Therefore, RV GLS values should be interpreted in a **modality-specific manner** and should not be directly compared across imaging techniques

#### ****Conclusion****

Three-dimensional echocardiography demonstrates a consistent association with CMR for the assessment of right ventricular volumes but modest underestimation of volumetric and functional parameters. Abnormal right ventricular strain was observed in patients with CMR-defined systolic dysfunction, supporting the clinical relevance of strain analysis. Overall, 3DE may serve as a feasible complementary tool for longitudinal right ventricular assessment in patients with rTOF, alongside CMR as the reference standard.

**Discussion**

**Page 14, lines 269-274**

**Page 14, lines 277-279**

**Pages 15, lines 288-293**

**Conclusion: page 3, lines 45-51; pages 19, lines 372-378**

Reviewer #2:

This study addresses a significant clinical issue in adult congenital heart disease (ACHD), specifically the onset of right ventricular (RV) systolic dysfunction following the repair of Tetralogy of Fallot. It introduces an alternative method for assessing RV volume and function in these patients, aside from the gold standard of cardiac magnetic resonance imaging (CMR). The research also compares the commonly used two-dimensional echocardiography (2DE) for evaluating RV volumes and myocardial deformation in patients with repaired Tetralogy of Fallot. The study met its power calculation requirements (29 required, 46 enrolled, 43 analyzed), which enhances the credibility of its findings. By defining RV dysfunction as an RV ejection fraction (RVEF) of less than 48%, the study establishes a clear threshold for diagnostic utility analysis. The use of Bland-Altman plots to evaluate bias and receiver operating characteristic (ROC) curves (area under the curve, AUC) to assess diagnostic accuracy is an appropriate statistical approach for a validation study. The writing maintains a professional tone and adheres to standard medical reporting. The results logically progress from baseline characteristics to direct comparisons of volumes, strain parameters, and ultimately to diagnostic performance.
The authors indicate that the study data cannot be publicly shared due to institutional regulations. However, data can be accessed through the Institutional Ethics Committee at the Faculty of Medicine, Chiang Mai University, for researchers who meet confidentiality criteria. This approach limits the availability of the study data.
The research was conducted between November 2023 and December 2024, but ethical approval was obtained from the Institutional Review Board at the Faculty of Medicine of Chiang Mai University in June 2025. Typically, ethical approval should precede data collection.

Patients with suboptimal 3D echocardiographic images (such as those with large RVs) as well as those with significant residual shunts and arrhythmias were excluded from the study. This exclusion is noteworthy, as these patients are crucial for integrating imaging modalities into clinical care and assessing the need for follow-up procedures like pulmonary valve replacements.
While the study's title indicates that it includes patients with repaired Tetralogy of Fallot, it is important to note that 32.6% of participants were only palliated. This subgroup may exhibit different pressure and volume dynamics affecting their right ventricles compared to fully repaired patients, given that surgical repair influences RV volume, function, and strain patterns. Furthermore, a correlation could have been better established between the timing of the study and the duration since repair.

Although CMR is generally more expensive and technically complex, three-dimensional echocardiography (3DE) may be more accessible and cost-effective. However, the study should address considerations regarding cost, required technical expertise, and the availability of 3DE software for broader implementation. Additionally, it would be beneficial to discuss the validity and applicability of TomTec Imaging Systems, which is used for this study, for assessing RV volume, function, and myocardial strains in non-cardiac patients.Finally, the authors acknowledge that 3DE tends to underestimate volumes compared to CMR. It would be useful to include a brief commentary on whether this underestimation is systematic (consistently lower) or random, as systemic bias could be corrected in clinical practice using an adjustment factor.

We sincerely thank the reviewer for the thoughtful and constructive overall evaluation of our work

- The research was conducted between November 2023 and December 2024, but ethical approval was obtained from the Institutional Review Board at the Faculty of Medicine of Chiang Mai University in June 2025. Typically, ethical approval should precede data collection.

**Answer:** Thank you for raising this important point regarding the timing of ethical approval. We would like to clarify that this study was designed as a **retrospective observational study.** The research involved analysis of **existing clinical data collected between November 2023 and December 2024,** prior to formal study conception. Ethical approval was therefore obtained from the Institutional Review Board of the Faculty of Medicine, Chiang Mai University, in **June 2025,** specifically to permit **retrospective access, analysis, and publication of these data.**

Importantly, the echocardiography and cardiac magnetic resonance imaging (CMR) examinations were performed as part of **routine clinical care**, not for research purposes. At our center, a standardized clinical protocol has been established whereby **patients undergoing CMR routinely receive echocardiography on the same day**, with the primary intention of optimizing patient care. This approach allows echocardiographic findings to serve as a practical reference for **subsequent clinical follow-up,** particularly in future visits when assessment may rely on clinical evaluation and echocardiography alone, without repeat CMR.

No additional imaging, interventions, or deviations from standard care were performed for the purpose of this research. All data were anonymized prior to analysis, and the study was conducted in full compliance with institutional and ethical regulations governing retrospective studies.

We have clarified this point in the **Ethical Approval** section of the revised manuscript.

“This study was conducted as a **retrospective observational study** using clinically acquired imaging data collected between November 2023 and December 2024. All echocardiography and cardiac magnetic resonance imaging examinations were performed as part of **routine clinical care** according to an institutional protocol. Ethical approval was obtained from the Institutional Review Board of the Faculty of Medicine, Chiang Mai University, to permit **retrospective access, analysis, and publication of the data.”**

**Methods and Ethical approval process and inform consent**

Page 6, line 112-117

- Patients with suboptimal 3D echocardiographic images (such as those with large RVs) as well as those with significant residual shunts and arrhythmias were excluded from the study. This exclusion is noteworthy, as these patients are crucial for integrating imaging modalities into clinical care and assessing the need for follow-up procedures like pulmonary valve replacements. While the study's title indicates that it includes patients with repaired Tetralogy of Fallot, it is important to note that 32.6% of participants were only palliated. This subgroup may exhibit different pressure and volume dynamics affecting their right ventricles compared to fully repaired patients, given that surgical repair influences RV volume, function, and strain patterns. Furthermore, a correlation could have been better established between the timing of the study and the duration since repair.

**Answer:** We agree that patients with markedly dilated right ventricles, residual shunts, and arrhythmias represent a clinically important subgroup, particularly with respect to decision-making for interventions such as pulmonary valve replacement. However, these exclusion criteria were applied to ensure technical validity and measurement reliability, as suboptimal three-dimensional echocardiographic image quality especially incomplete RV coverage in severely dilated ventricles can lead to inaccurate volumetric and strain measurements. We have clarified this rationale in the Study Limitations section and acknowledge that our findings may not be directly generalizable to patients with very advanced RV remodeling or complex residual lesions. In this context, cardiac magnetic resonance imaging remains the reference standard for accurate evaluation of right ventricular size and systolic function, particularly in patients with advanced disease or complex anatomy. **Nevertheless, our findings provide clinically relevant insights into the performance of three-dimensional echocardiography in a well-defined and commonly encountered rTOF population, supporting its role as a complementary modality for routine follow-up.**

Regarding the inclusion of patients who had undergone palliative procedures, we would like to clarify that all patients in this cohort had a final anatomical diagnosis of repaired Tetralogy of Fallot, and the term “palliated” refers to patients who underwent initial staged palliation prior to definitive surgical repair. These patients subsequently received complete repair and were included in the analysis as part of the repaired TOF population. We agree that staged and primary repair strategies may influence RV loading conditions and myocardial mechanics; however, the present study was not powered to perform subgroup analyses based on surgical strategy. This has now been acknowledged as a limitation. **Despite this, the inclusion of patients with varied surgical pathways reflects real-world clinical practice and enhances the external relevance of our findings within the repaired TOF population.**

We also agree that the interval between definitive repair and imaging may influence RV size, function, and strain patterns. Although the duration since repair was recorded, the relatively modest sample size limited our ability to perform robust correlation or subgroup analyses based on time since repair. We have now explicitly addressed this point in the Study Limitations sections and suggest that future larger, longitudinal studies are needed to evaluate the impact of surgical timing and repair strategy on RV remodeling and functional parameters. **Nonetheless, the standardized same-day imaging protocol in our study minimizes physiologic variability and strengthens the internal validity of the observed associations.**

We appreciate the reviewer’s insightful comments, which have helped us better contextualize the applicability and limitations of our findings in clinical practice

#### “**Study Limitations**

Several limitations of this study should be acknowledged. First, the relatively modest sample size may limit the generalizability of our findings and precluded robust subgroup analyses, including stratification by surgical strategy or duration since definitive repair. Second, image quality remains a challenge for 3DE, particularly in patients with suboptimal acoustic windows or marked right ventricular dilation. In such cases, incomplete right ventricular coverage within a single acquisition may lead to systematic underestimation of volumetric and functional parameters, reducing measurement reliability. Consequently, patients with very advanced right ventricular remodeling, significant residual shunts, or arrhythmias were excluded, and our findings may not be directly applicable to these clinically important subgroups, for whom CMR imaging remains the reference standard for accurate assessment of right ventricular size and systolic function. Third, echocardiographic strain measurements may be influenced by vendor-specific analysis algorithms, which could affect reproducibility across platforms. In addition, the use of a single experienced assessor may raise concerns regarding potential observer bias, although standardized post-processing protocols and blinding between echocardiographic and CMR analyses were applied to mitigate this risk. Furthermore, only moderate correlations were observed between 2D and 3D strain parameters, highlighting modality-specific differences and underscoring the importance of interpreting strain values within the context of the imaging technique used. Despite these limitations, the study reflects real-world clinical practice and benefits from standardized same-day echocardiography and CMR acquisition, minimizing physiologic variability. Future larger, multicenter studies with multiple independent readers are warranted to validate the reproducibility of 3DE-derived parameters and to further define their role in risk stratification and long-term outcome assessment in patients with rTOF”.

**Study Limitations**

**Pages 18 and 19, Line 249-373**

- Although CMR is generally more expensive and technically complex, three-dimensional echocardiography (3DE) may be more accessible and cost-effective. However, the study should address considerations regarding cost, required technical expertise, and the availability of 3DE software for broader implementation. Additionally, it would be beneficial to discuss the validity and applicability of TomTec Imaging Systems, which is used for this study, for assessing RV volume, function, and myocardial strains in non-cardiac patients.Finally, the authors acknowledge that 3DE tends to underestimate volumes compared to CMR. It would be useful to include a brief commentary on whether this underestimation is systematic (consistently lower) or random, as systemic bias could be corrected in clinical practice using an adjustment factor.

**Answer:** Thank you for this comprehensive and constructive comment.

We agree that broader implementation of three-dimensional echocardiography (3DE) requires consideration of **cost, technical expertise, and software availability.** While 3DE is generally less expensive and more accessible than cardiac magnetic resonance (CMR), its use does require dedicated hardware, vendor-specific software, and trained operators. We have expanded the Discussion to clarify that the clinical applicability of 3DE is most relevant in centers with established echocardiographic expertise and access to validated 3D analysis software, and that appropriate training is essential to ensure reliable measurements.

Regarding the use of **TomTec Imaging Systems,** this platform has been widely validated for right ventricular volume, function, and myocardial strain assessment in patients with congenital and acquired heart disease. Although our study focused on patients with repaired Tetralogy of Fallot, prior studies have demonstrated the feasibility and validity of TomTec-based 3DE analysis across diverse patient populations, including non-congenital cardiac conditions. We have clarified that our findings should be interpreted within the context of this validated software environment, and that results may not be directly interchangeable with those obtained using other vendor-specific platforms.

Finally, we agree that the tendency of 3DE to underestimate right ventricular volumes compared with CMR warrants clarification. In our study, the underestimation was **systematic rather than random,** as evidenced by a consistent negative mean bias on Bland Altman analysis. This systematic bias is consistent with prior literature and is likely related to challenges in endocardial border delineation and incomplete right ventricular coverage in echocardiography. While such a bias theoretically could be addressed using correction or adjustment factors, we did not apply such corrections in this study to preserve methodological transparency and comparability with existing literature. We have now added a brief discussion noting that future studies may explore standardized correction approaches to enhance the clinical utility of 3DE-derived volumetric measurements.

We appreciate the reviewer’s insightful comments, which have helped us strengthen the discussion regarding the practical implementation and interpretation of three-dimensional echocardiography in clinical practice.

“Although 3DE is generally more accessible and cost-effective than CMR, its broader clinical implementation depends on equipment availability, validated analysis software, and operator expertise. Accurate acquisition and post-processing require trained personnel and vendor-specific platforms. In this study, right ventricular volumetric and strain analyses were performed using TomTec Imaging Systems, which has been widely validated for assessment of right ventricular size, function, and myocardial deformation in both congenital and non-congenital cardiac populations; however, measurements may not be directly interchangeable across different software platforms. Consistent with prior reports, 3DE demonstrated a tendency to underestimate right ventricular volumes compared with CMR. In our study, this underestimation was systematic rather than random, as indicated by a consistent negative mean bias on Bland Altman analysis, likely related to challenges in endocardial border delineation and incomplete right ventricular coverage inherent to echocardiographic imaging”.

Page 17, lines 326-337

6. PLOS authors have the option to publish the peer review history of their article ([what does this mean?](https://track.editorialmanager.com/CL0/https:%2F%2Fjournals.plos.org%2Fplosone%2Fs%2Feditorial-and-peer-review-process%23loc-peer-review-history/1/010f019bb203d5af-7764a7ea-c740-471f-bfbe-4f7befac29fd-000000/XMwHmScu7Ylswwe53NNWsbjmNzvlRdhVzcpGj1dwI5s=245)). If published, this will include your full peer review and any attached files.

If you choose “no”, your identity will remain anonymous but your review may still be made public.

Response to reviewer on 30/1/2026

1. Thank you for your response. However, the concern of the journal office is not the MRI images, but rather the clip art imagery used to construct Figure 1. This includes the clip art of the crowd of people, and other images. Please clarify the source of these artistic depictions and their copyright status.

**Answer:** Thank you very much for this important clarification.

We sincerely apologize for the misunderstanding. We understand that the concern relates specifically to the clip art elements used in the original Figure 1 (including the crowd illustration and other artistic components)

In response to this concern, we have completely removed the previous version of Figure 1 and have created a new figure to replace it. The revised Figure 1 has been fully reconstructed by the authors using original schematic designs, without incorporating any third-party clip art, stock images, or copyrighted artistic materials.

The new figure is entirely author-generated and does not contain any copyrighted visual elements from external sources. Therefore, there are no copyright restrictions associated with the revised figure.

We appreciate the journal office’s careful review and thank you for giving us the opportunity to correct this issue.
